# Correction: Absence of pathogenic mutations in CD59 in chronic inflammatory demyelinating polyradiculoneuropathy

**DOI:** 10.1371/journal.pone.0215784

**Published:** 2019-04-17

**Authors:** Lena Duchateau, Lorena Martín-Aguilar, Cinta Lleixà, Andrea Cortese, Oriol Dols-Icardo, Laura Cervera-Carles, Elba Pascual-Goñi, Jordi Diaz-Manera, Ilaria Callegari, Diego Franciotta, Ricard Rojas-Garcia, Isabel Illa, Jordi Clarimon, Luis Querol

The ninth author’s name is spelled incorrectly. The correct name is: Ilaria Callegari.
